# Blood Lead Levels among Blood Donors and High-Risk Occupational Groups in a Mining Area in Ghana: Implications for Blood Transfusion among Vulnerable Populations

**DOI:** 10.1155/2020/6718985

**Published:** 2020-07-10

**Authors:** Veronica Agyemang, Joseph K. Acquaye, Samuel B. E. Harrison, Felix B. Oppong, Stephany Gyaase, Kwaku P. Asante, Edeghonghon Olayemi

**Affiliations:** ^1^Kintampo Health Research Centre, Kintampo, Ghana; ^2^Department of Haematology, University of Ghana Medical School, Korle Bu, Accra, Ghana

## Abstract

Lead poisoning has been a major global health problem for decades, and blood transfusion has been suspected as a neglected potential source of lead exposure. Children and pregnant women are most vulnerable to the toxic effects of lead and over 40 percent of blood transfused in Ghana is given to children under 5 years. However, there is little data on the levels of lead in donor blood and the main sources of lead exposure in the Ghanaian population. This study compared blood lead levels (BLL) among selected occupations at risk of lead exposure with healthy blood donors in nonexposed occupations in a Ghanaian mining area. We enrolled 40 participants each from the following high-risk occupational groups: small scale miners, painters/sprayers, drivers/fuel station attendants, and auto-mechanics as well as 40 healthy blood donors (made up of teachers, traders, and office workers). One millilitre of blood was collected from each participant for determination of their BLL, haemoglobin concentration, and blood film morphology. A total of 200 participants made up of 186 (93%) males and 14 (7%) females were enrolled. The mean age of participants was 28.6 ± 8.2 years and their geometric mean (GM) BLL was 6.3 GSD 1.4 *µ*g/dL [95% CI: 6.0 – 6.6]. Participants in high risk occupations had significantly higher GM BLL of 6.7 *µ*g/dL [95% CI :6.4−7.0] compared to 5.0 *µ*g/dL [95% CI: 4.4−5.7] for healthy blood donors [*p* < 0.001]. The prevalence of elevated BLL (≥5 *µ*g/dL) among the entire study participants, high risk occupations and blood donors was 84.5%, 89.4% and 65% respectively. There was significant association between elevated BLLs and working in an at-risk occupational group [aOR = 3.58, *p* = 0.014]. Haemoglobin concentration was not significantly associated with elevated BLLs. Basophilic stippling was not observed in any of the blood smears. Blood lead levels were high in blood donors and at-risk occupations in the study area and occupation was associated with elevated BLLs. It is important that measures to safeguard the integrity of donor blood go beyond screening for infectious diseases to include screening individuals in high-risk occupations for lead and other heavy metals to ensure that donor blood from such individuals is safe and does not pose potential danger to the health of vulnerable populations such as children and pregnant women.

## 1. Introduction

Globally, lead poisoning is a major public health challenge, and work-related exposure to lead is an important source of lead poisoning in adults [1]. The various uses of lead led to widespread environmental contamination and human exposure in various parts of the world. Exposure to lead may occur via inhalation of lead particles, consumption of food or water contaminated with lead, and through direct skin contact with contaminated objects [2, 3]. Vital sources of lead exposure in the environment include vehicle assembly plants, mining activities, battery recycling activities, manufacturing, smelting, sustained use of lead-containing paint, and leaded gasoline [3]. More than 75% of the global lead consumption is in lead-acid battery production [2, 3].

Worldwide, lead exposure contributes to about 600,000 new cases of children with intellectual disabilities and 143,000 deaths per year [3]. In the United States, over 3 million people are exposed to lead at their workplaces [3].

Previously, lead poisoning was defined as a venous blood lead level (BLL) of 10 *μ*g/dl or higher [4]. However, the Centers for Disease Control and Prevention (CDC) and the World Health Organisation (WHO) have recently adopted a series of recommendations indicating that “there is no safe level of blood lead in children” [3]. The CDC therefore recommended that BLL ≥ 5 *µ*g/dL is considered elevated [2]. These recommendations, however, do not address strategies to avoid lead exposure occurring via blood transfusion, even though transfused lead is substantially more bio-available than oral lead and a dose-response relationship exists between the lead concentration of transfused packed red blood cells and post-transfusion BLLs among premature infants [5]. Adverse effects of lead have been reported in adults at BLLs that were initially thought to be harmless. These include impaired renal function, essential tremor, and hypertension at BLL less than 10 *μ*g/dL [3].

In acute situations, exposure to high levels of lead may result in vomiting, headaches, abdominal pain, tremors, convulsions, muscle weakness, coma, and even death in adults [6], while prolonged exposure to low levels of lead has been associated with reduced intelligence quotient (IQ) and neurobehavioral problems in children [6, 7]. Lead also inhibits normal haem synthesis resulting in a characteristic microcytic, hypochromic haemolytic anaemia [7].

Blood transfusion is a hidden source of lead exposure [8]. Since over 40% of blood transfused in Ghana is given to children under the age of 5 years [9] who are particularly vulnerable to the toxic effects of lead, it is important to ensure that donated blood is not contaminated by lead. By studying populations that are prone to lead exposure, critical decisions can be made about their lead levels such as targeted educational campaigns, deferring them from blood donation and strict adherence to protective measures to reduce their lead exposure to ensure that donor blood from such individuals is safe and does not pose potential threat to the health of vulnerable populations such as children and pregnant women. The primary aim of this study was to determine BLLs among selected at-risk occupational groups and compare with blood donors in nonexposed occupations, living and working in a mining area in Ghana.

## 2. Materials and Methods

**Figure 1:**
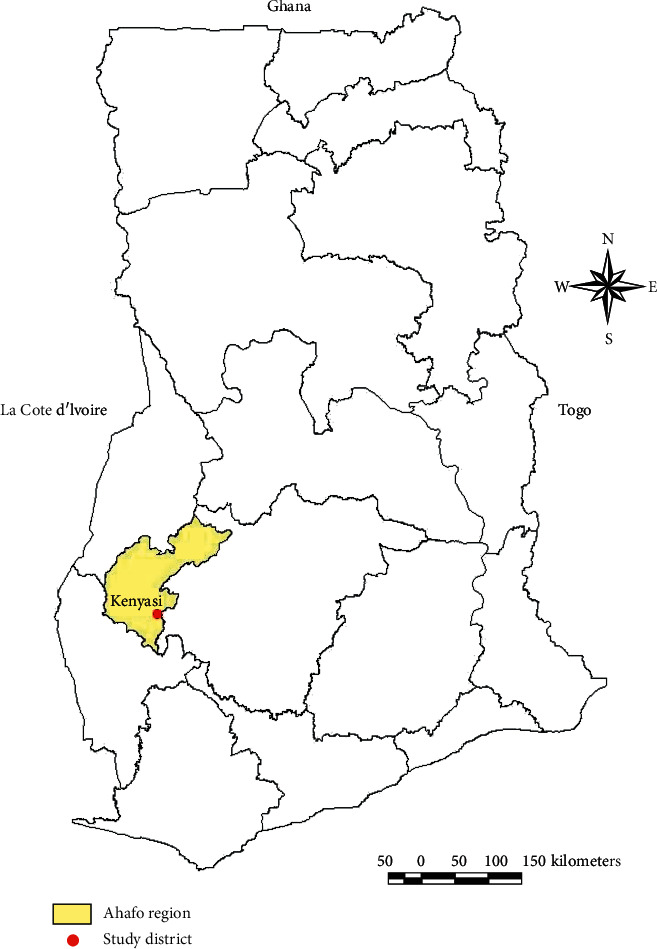
Map of Ghana showing Kenyasi.

This cross-sectional comparative study was carried out between 2^nd^ January 2017 and 31^st^ July 2017 among four distinct at-risk occupational groups and blood donors not involved in any at-risk occupation at Kenyasi in the Ahafo region of Ghana. Kenyasi is a gold mining town located in Asutifi North district of the Ahafo region of Ghana. Kenyasi (Figure 1) is the capital town of the district with a population of 52,259 [10]. Non exposed healthy blood donors serving as controls were recruited from Hwidiem St. Elizabeth Hospital which is the main hospital in the district with a functional blood bank serving the entire population of Kenyasi and its environs. The hospital on average receives about 100 blood donations per month. Laboratory analyses were carried out at the Kintampo Health Research Centre (KHRC) laboratory where full blood count and blood smears were done and the chemistry laboratory of the Ghana Atomic Energy Commission for blood lead measurements.

## 3. Study Population

The study population was made up of individuals from among four at-risk occupations, namely, auto mechanics, fuel station attendants/automobile drivers, painters/sprayers, and small-scale miners who met the eligibility criteria. The control group was drawn from among apparently healthy blood donors at Hwidiem St. Elizabeth Hospital, who did not belong to any of the selected at-risk occupations, they were mainly office workers, traders, and teachers, but they were not preselected. Inclusion and exclusion criteria: adults between 18 to 60 years, apparently healthy, who have not been permanently deferred from blood donation and who were willing to provide written informed consent were eligible for enrolment into the study. Individuals who were on medication for any known chronic disease were excluded.

## 4. Sample Size

The sample size for this study was determined by assuming a mean BLL (*µ*g/dL) of 8.5, 8.5, 6.5, 6.5 and 5.0 for small scale miners, auto-mechanics, painters/sprayers, fuel attendants/drivers and blood donors respectively. This is based on the recommendation by the CDC that BLL ≥5 *µ*g/dL is elevated [2]. With a common standard deviation of 4.5, a 5% type I error rate and a power of 90%, an estimated sample size of 180 made up of 36 participants each was required. Adjusting for a 10% nonresponse rate in each group, a total sample size of 200 that is, 40 in each group was required to compare the differences in their BLLs as well as some predisposing factors. The formula is shown as follows:

(1) $$\begin{array}{c} n=2\frac{\left(\sigma Z_{1-\left(\alpha /2\tau \right)}+Z_{1-\;\beta }\right)2}{\mu_{A}-\;\mu_{B}}, \end{array}$$

where *n* is the sample size, *σ* is the standard deviation, *α* is the type I error rate, *τ* is the number of comparisons, *β* is the type II error, meaning 1 − *β* is the power, *μ* is the estimated mean lead concentration for a particular occupational group, *Z* is the standard normal distribution, and *A* and *B* refer to any of the occupational groups.

### 4.1. Data and Sample Collection

All healthy individuals within each of the selected at-risk occupational groups and all blood donors between ages 18 and 60 years who gave informed consent were conveniently recruited into the study. After completing a questionnaire with data such as age, sex, occupation, and source of drinking water, 1 mL of venous blood was drawn, 500 *μ*L of which was dispensed into two EDTA tubes. The samples collected were preserved at 2 to 8°C in an ice chest on-site. One tube was sent to the Kintampo Health Research Centre (for full blood count and blood smear preparation) and the other to the Ghana Atomic Energy chemistry laboratory (for blood lead analysis). Haemoglobin measurement was done using the ABX Micros 60 Haematology Analyzer (Montpellier, France) following the manufacturers' instruction. Three levels of controls (low, normal, and high) were done daily before samples were analysed. Peripheral blood smears were prepared and stained with Leishman stain as previously described [11].

## 5. Measurement of Blood Lead Levels

Blood lead concentrations were measured with Varian AA 240FS—atomic absorption spectrometer in acetylene-air flame (procedure adopted from Varian publication no. 85-100009-00, revised 1989). Doubled distilled water was used for blanking. Three standard concentrations of 2.0 mg/L, 5.0 mg/L, and 10.0 mg/L were used. For every ten samples analysed, a blank, one quality control sample, and two standards were run. 

## 6. Data Management

Unique identification numbers were assigned to each person instead of names to ensure confidentiality. All data were coded and entered into Microsoft Excel 2010 and then transferred into STATA version 14.0 for analysis.

## 7. Data Analysis

The distribution of the BLL was checked by both graphical method (using a histogram of BLLs) and Shapiro–Wilk test for normality. Both indicated non normality of the BLLs. Basic descriptive statistics were performed on all important variables. Means and ranges were presented for all continuous variables, whereas frequencies were presented for all categorical variables. Since the distribution of the blood lead levels showed non normality, geometric mean and its 95% confidence interval were used to describe participant's BLLs. Kruskal–Wallis H test was used to test for differences in means of BLLs among the four different occupational groups as well as the blood donors. Participant's BLLs were then categorized into a binary outcome based on CDC recommendations that BLL ≥5 *μ*g/dL defined elevated blood lead level (EBLL). This binary indicator, EBLL, was then used as the response variable and participant's occupation as the main predictor variable. Participants were also divided into two groups by their oocupation (which was the main predictor variable): high-risk occupational group (small-scale miners, fuel attendants/drivers, auto-mechanics, and painters/sprayers) and low-risk oocupational group (blood donors). Univariate logistic regression was then fitted to assess the relationship between the response variable and the main predictor variables as well as other factors. These factors include participant's haemoglobin concentration, age, sex, marital status, number of years of working in that occupation, source of drinking water, cooking with gas or not, and educational level. Factors found to be statistically significant at 0.2 level of significance including participants' occupation were adjusted for in the multiple logistic regression. All statistical tests in the multiple logistic models were two-sided and at a significance level of 0.05. 

## 8. Ethical Approval

Ethical approval was obtained from the Ethical and Protocol Review Committee of the College of Health Sciences, University of Ghana (CHS-Et/M.8-P4.1/2016-2017) as well as from the Institutional Ethics Committee of the Kintampo Health Research Centre (KHRCHC/2017.2). Permission was also sought from the leaders of occupational groups involved in the study and from the head of the blood bank at St. Elizabeth Hospital, Hwidiem.

## 9. Results

### 9.1. Demographic Characteristics

A total of 200 participants, made up of 186 (93%) males and 14 (7%) females, were enrolled. The mean age of participants was 28.6 ± 8.2 years [95% CI: 27.4 – 29.7] ranging from 18 to 57 years. Majority (56%) of the participants were unmarried and their highest level of education was up to the junior high/secondary school level. Sachet water and pipe-borne water were the main sources of drinking water within the study area. Participants' mean haemoglobin concentration was 14.1 g/dl ± 1.6 g/dl ranging from 7.4 g/dl to 16.8 g/dl. On average, participants had worked for 5.0 years ± 5.4 years in their respective occupations. This ranged from less than one year to 35 years.  Table 1 gives the detailed description of participants' basic demographics.

The mean haemoglobin concentrations among the four at-risk occupations were comparable. In all, blood donors had the highest average number of years of being employed, and they also had the oldest participant who was 57 years (Table 2).

### 9.2. Blood Lead Level

The geometric mean (GM) blood lead level (BLL) of the entire study participants was 6.3 *μ*g/dL (95% CI: 6.0–6.6) with a minimum BLL of 1.8 *μ*g/dL and a maximum of 14.4 *μ*g/dL. The GM BLL for the exposed group was 6.7 *μ*g/dL (95% CI: 6.4 *µ*g/dL–7.0 *μ*g/dL), and for the nonexposed group, it was 5.0 *μ*g/dL (95% CI: 4.4 *μ*g/dL–5.7 *μ*g/dL). Auto mechanics recorded the highest geometric mean BLL of 7.9 *μ*g/dL, while blood donors recorded the lowest geometric mean BLL of 5.0 *μ*g/dL (Table 3).

There was a statistically significant difference in the mean blood lead levels of various occupational groups (*χ*^2^ (4) = 199.0; *p*=0.001).

### 9.3. Elevated Blood Lead Level (EBLL)

The prevalence of EBLL among the entire study participants was 84.5% (169/200). EBLLs among exposed occupational groups and blood donors were 89.4% and 65%, respectively. The highest prevalence was in painters/sprayers (100%) followed by auto mechanics 97%. The least prevalence was recorded in fuel attendants and blood donors with the same prevalence of 65%. There was a statistically significant association between EBLLs and respondent's occupational group (Fisher's exact *p* < 0.001) Table 4).

Univariate exact logistic regression analysis was fitted to assess factors influencing EBLL and factors such as occupational group, age, sex, drinking pipe borne/bore hole water, and marital status were found to be statistically associated with EBLLs. Those factors found to be significant in the univariate model were included in the final multiple logistic regression model. After adjusting for all possible confounders (age, sex, educational level, marital status, and source of drinking water), the significant predictor for EBLL in the study area was occupation [aOR = 3.58, *p* = 0.014]. Specifically, auto-mechanics, painters/sprayers and small scale-miners had significantly EBLLs (Table 5).

Blood smears did not show any basophilic stippling.

## 10. Discussion

The aim of this study was to determine the BLLs among some selected at-risk occupations and compare them with the BLLs of blood donors who do not work in any known lead exposed occupations in a mining area in Ghana. 

The geometric mean BLL for participants in the study was 6.3 *μ*g/dL (95% CI: 6.0–6.6). It was 5.0 *μ*g/dL (95% CI: 4.4–5.7), 6.8 *μ*g/dL (95% CI: 6.3–7.3), 5.2 *μ*g/dL (95% CI: 4.7–5.8), 7.9 *μ*g/dL (95% CI: 7.2–8.6), and 7.2 *μ*g/dL (95% CI: 6.9–7.7) among the blood donors, small-scale miners, fuel attendants/drivers, auto mechanics, and painters/sprayers, respectively. These values are higher than <5 *μ*g/dL recommended by the CDC [2]. The prevalence of EBLL among the study participants was 84.5%. Occupation was a significant predictor of EBLL in our study, a finding that is supported by several other studies that associated EBLLs and lead poisoning with occupational exposure [1, 12, 13]. Blood transfusion has been considered a possible source of lead exposure [5, 14], it is worrying that in this study, the prevalence of EBLL for apparently healthy blood donors was 65%. The mean BLLs recorded in blood donors in Egypt, Morocco, and Italy were, however, higher than in this study being 17.0 *μ*g/dL, 8.7 *μ*g/dL, and 14.8 *μ*g/dL, respectively, compared to 1.0 *μ*g/dL in the blood banks in the USA [15, 16]. The mean BLLs of taxi drivers and mechanics in Jordan were 28.8 *μ*g/dL and 24.6 *μ*g/dl, respectively [17], also higher than in our study. In Nigeria, auto mechanics recorded a far higher mean BLL of 36.1 *μ*g/dL [18]. Transfusing blood with such a high lead concentration could have severe health implications on vulnerable populations such as neonates and children.

The BLL of fuel attendants/drivers was comparable with healthy blood donors (Table 3). The phasing out of leaded gasoline since 2004 in Ghana [19] may have contributed to the relatively low BLL in fuel attendants/drivers. However, this was still higher than the level recommended by the CDC. It is possible that during the gold mining process, small scale miners encounter lead which forms part of the earth's crust. The continued use of lead containing chemicals during mining may have predisposed small scale miners to high lead levels. These chemicals could also cause background environmental contamination which could affect the soil and water bodies. This could explain the exposure to lead in healthy blood donors in the area. The use of lead acid batteries by auto mechanics and handling of lead containing spare parts may explain the EBLLs found in them. Their activities may also contribute to background soil contamination which may serve as a source of lead exposure to inhabitants.

The fact that all painters/sprayers recorded significantly elevated BLL raises suspicion about the persisting use of lead in paints in the area and perhaps other parts of the country. Similar studies in Iranian painters recorded a mean of 4.7 *µ*g/dl [20]. Interestingly, we did not come across any published data on the sources of lead exposure in the study area though studies in other parts of Ghana have demonstrated lead contamination of soil from industrial activities [19]. It is important that attention is given to neglected sources of lead exposure as BLLs in the range recorded in this study have been associated with impaired renal function, increased risk for hypertension, and essential tremor among adults [21] and sub-clinical neurological toxicities in younger children who are exposed to BLLs as low as 1–3 *μ*g/dL [22].

Haemoglobin concentration was not significantly associated with EBLLs (Table 5). This could be explained by the fact that the BLLs recorded among our participants were lower than the levels at which haem synthesis is impaired by lead. Studies have shown that haem synthesis does not decrease until the activity of *δ*-ALAD is inhibited by 80–90%, and this occurs at a BLL ≥ 50 *μ*g/dL [23, 24].

Basophilic stippling is another important haematological indicator of lead poisoning. However, blood smears prepared in this study did not show any basophilic stippling because basophilic stippling does not appear in red cells until BLL is ≥ 50 *μ*g/dL for adults and ≥40 *μ*g/dL for children [7, 25].

## 11. Conclusion

Blood lead levels are high in blood donors and occupational groups in the study area and occupation is associated with elevated BLLs. It is important to enforce occupational laws to reduce occupational exposure to lead. Atypical sources of lead exposure, such as blood transfusion, deserve attention since donor blood could be a major hidden source of lead exposure, especially among vulnerable populations. 

 Replacement blood donation by family and friends is common in the Ghanaian society, and therefore, individuals in these high-risk occupations are potential blood donors. It is important that measures to ensure the safety of donor blood go beyond screening for only infectious diseases. Perhaps, policy formulation to include screening individuals in high-risk occupations for heavy metals may be lifesaving. 

## Figures and Tables

**Table 1:** Basic demographic characteristics.

Variable		*N* (%)
Sex	Male	186 (93.0)
	Female	14 (7.0)

Marital status	Married	88 (44.0)
	Unmarried	112 (56.0)

Educational level	No formal education	2 (1.0)
	Primary education	23 (11.5)
	Middle/junior high school	113 (56.5)
	Secondary education	43 (21.5)
	Tertiary education	19 (9.5)

Source of drinking water^∗∗^	Pure/sachet water	194 (97.0)
	Pipe borne water	149 (74.5)
	Well/borehole	65 (32.5)

Building material	Cement with roofing sheets	198 (99.0)
	Mud with roofing sheet	2 (1.0)

Gas for cooking	Yes	139 (69.5)
	No	61 (30.5)

**^∗∗^**Study participants had multiple sources of drinking water.

**Table 2:** Basic variable description by occupation.

Variable	Occupation	Observation	Mean	Range
Haemoglobin	Blood donors	40	13.7	12.1–16.1
	Small scale miners	40	14.6	10.9–16.8
	Fuel attendants/drivers	40	14.3	11.5–16.8
	Auto-mechanics	40	14.1	10.0–15.7
	Painters/sprayers	40	14.0	7.4–16.1

Age	Blood donors	40	32.0	18.0–57.0
	Small scale miners	40	29.0	19.0–42.0
	Fuel attendants/drivers	40	29.3	20.0–47.0
	Auto-mechanics	40	25.0	18.0–40.0
	Painters/sprayers	40	27.0	18.0–55.0

Number of years employed	Blood donors	40	6.7	<1.0–35.0
	Small scale miners	40	5.5	1.0–15.0
	Fuel attendants/drivers	40	4.5	<1.0–22.0
	Auto-mechanics	40	3.9	<1.0–20.0
	Painters/sprayers	40	3.6	<1.0–15.0

**Table 3:** Distribution of blood lead level among occupational groups.

Occupation	Geometric mean	GSD∗	95% CI
Blood donors	5.0	1.8	4.4–5.7
Small scale miners	6.8	1.5	6.3–7.3
Fuel attendants/drivers	5.2	1.6	4.7–5.8
Auto-mechanics	7.9	1.8	7.2–8.6
Painters/sprayers	7.3	1.3	6.9–7.7

∗GSD is geometric standard deviation.

**Table 4:** Prevalence of elevated blood lead level among the occupational groups.

Occupation	*N*	Normal BLL *N* (%)	Elevated BLL *N* (%)	*P* value
Blood donors	40	14 (35.0)	26 (65.0)	
Small scale miners	40	2 (5.0)	38 (95.0)	
Fuel attendants/drivers	40	14 (35.0)	26 (65.0)	
Auto-mechanics	40	1 (2.5)	39 (97.5)	
Painters/sprayers	40	0 (0.0)	40 (100)	
Total	200	31 (15.5)	169 (84.5)	<0.001

**Table 5:** Logistic regression model showing factors influencing elevated blood lead levels.

Variable		OR (80% CI)	*P* value^1^	aOR (95% CI)	*P* value^2^
Sex	Female	1.0			
	Male	1.1 (0.8–1.4)	0.3057		

Age group (years)	18–26	1.0			
	27–35	0.6 (0.3–1.1)	0.3050	0.18 (0.3–2.5)	0.682
	36-44	0.5 (0.2–0.9)	0.1500	0.76 (0.2–3.6)	0.733
	>45	0.3 (0.1–0.8)	0.105	0.68 (0.1–4.6)	0.700

Haemoglobin concentration < 11.0g/dl	No	1.0			
	Yes	1.2 (0.9–1.3)	0.2208		

Occupational group	Blood donors	1.0			
	Fuel attendants	0.3 (0.1–0.5)	0.2978	0.3 (0.1–0.7)	0.4814
	Small scale miners	2.7 (0.9–11.4)	0.0046	2.2 (0.5–21.6)	0.0086
	Auto-mechanics	Predicts elevated BLL			
	Painters/sprayers	Predicts elevated BLL perfectly			

Occupation (unexposed versus exposed)	Blood donors	1.0			
	Exposed occupations	4.53 (2.64–7.75)	<0.001	3.58 (1.29–9.95)	0.014

Marital status	Married	1.0			
	Unmarried	2.0 (1.2–3.3)	0.090	1.60 (0.5–5.3)	0.4370

Pipe borne water	No	1.0			
	Yes	2.1 (1.1–3.8)	0.1139	1.6 (0.6–3.9)	0.3860

Years employed	<1 year	1.0			
	1–10 years	0.5 (0.1–2.0)	0.5340		
	> 10 years	0.8 (0.1–3.9)	0.8240		

Well/borehole	No	1.0			
	Yes	1.5 (0.8–2.3)	0.3890		

Cooking with gas	No	1.0			
	Yes	0.5 (0.2–1.0)	0.2048	0.7 (0.2−1.9)	0.6053

Where OR is the unadjusted odds ratio and aOR is the adjusted odds ratio

## Data Availability

The data supporting the findings of this study are available to researchers who meet the criteria for access to confidential data from the corresponding author upon request. This will be done after approval of the request by the Ethics Committee of the College of Health Sciences, University of Ghana, and the Kintampo Health Research Centre.
